# Actinomyces Colonization in Keratocystic Odontogenic Tumor: A Case Report

**Published:** 2015-06

**Authors:** Zohreh Jafari-Ashkavandi, Fereshteh Kamali

**Affiliations:** 1 Dept. of Oral and Maxillofacial Pathology, School of Dentistry, Shiraz University of Medical Science, Shiraz, Iran;; 2 Postgraduate Student, Dept. of Oral and Maxillofacial pathology, School of Dentistry, Shiraz University of Medical Science, Shiraz, Iran;

**Keywords:** Actinomycosis, Keratocystic Odontogenic Tumor, Odontogenic Cyst, Surgical Treatment

## Abstract

Actinomycosis is an anaerobic infection that involves the craniofacial region and its colonization has rarely been reported in the developmental odontogenic cysts. In the present report, a case of odontogenic keratocyst (which is now called keratocystic odontogenic tumor) with the colonization of actinomyces is introduced and its significance is discussed.

## Introduction

Actinomyces is an anaerobic, gram-positive and filamentous bacterium which is found in normal oral flora.[1-2] It is not pathogenic when present on intact mucosa, however, it may cause infection when penetrating the underlying connective tissue.[2-3] Actinomyces infection is generally classified according to the organ or area of involvement.[4-5] The craniofacial infection caused by this pathogen accounts for 60% of all cases.[1]

In healthy people, the bacteria may be colonized in tonsillar crypts, dental plaque, calculus, nonvital teeth, gingival sulcus , and periodontal pocket.[3] The organism may invade tissue through a nonvital tooth or a trauma such as tooth extraction[5] and leads to an acute or chronic infection. It may cause a suppurative infection in the periapical area even after a standard root canal therapy. Moreover, this pathogen may cause actinomycotic osteomyelitis with or without overlying soft tissue involvement,[4] granuloma or multiple sinus tracts. The infection is usually found in patients aged 20-29 years with male predominance.[2-6]

There have been many reports of actinomycosis infection in association with radicular cysts.[5] Besides, some cases of actinomycosis infection in dentigerous cyst have been reported without significant clinical changes.[6] To the best of our knowledge, colonization of bacteria in other developmental cystic lesions of the jaws such as odontogenic keratocyst which is now called keratocystic odontogenic tumor (KCOT) has been observed in one case of an infected odontogenic keratocyst without any description, in1975.[7-8] In the present study, we describe the clinical and radiographic features of a patient with actinomycosis colonization in KCOT wall.

## Case report

A 17 year-old male patient referred with chief complaint of swelling on right side of his face since 4 months ago. The patient had a history of surgical treatment for KCOT in left and right sides of maxilla and mandible 4 years ago. Clinical examination revealed a swelling on the right side of mandible with intact mucosa. No defect was observed in any other organs and the patient did not show any signs of Gorlin syndrome.

**Figure 1:**
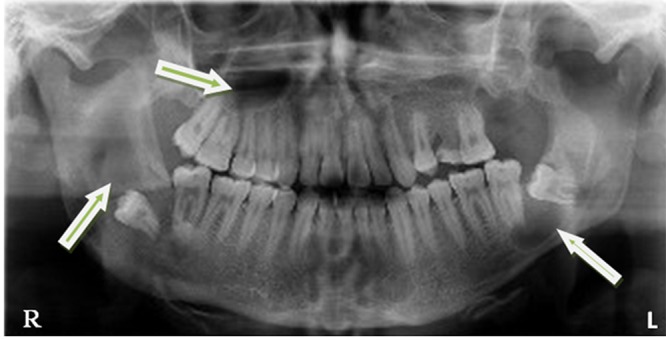
Panoramic view showing multiple radiolucent lesions in the mandible and maxilla (arrows)

Radiographs revealed multiple well-defined radiolucent lesions in the posterior mandible and left side of the maxilla. The mandibular lesions were associated with the crown of impacted third molars without any evidence of root resorption (Figure 1).

Excisional surgery of right and left mandibular lesions was performed under general anesthesia. The patient received routine postoperative treatment without any antibiotic therapy.

**Figure 2:**
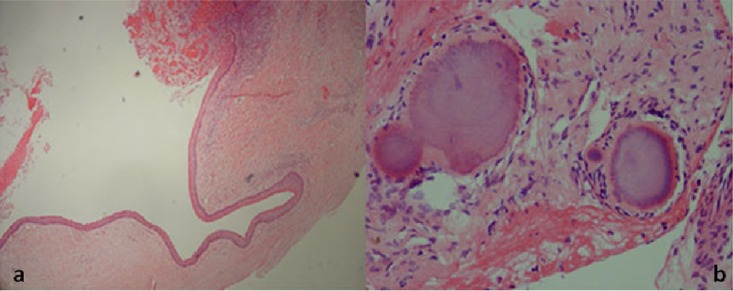
a: The cystic wall of KCOT   b: Typical colonies of actinomycosis without significant inflammation or fibrosis

Histopathologic examination revealed a cystic lesion lined by parakeratinized stratified squamous epithelium with palisaded basal layer (Figure 2a). The underlying fibrous connective tissue demonstrated severe inflammatory cell infiltration and a few bacterial colonies. The microbial colonies were irregular-shaped, round or lobulated, formed by a radiating mass of basophilic mycelia and club-shaped eosinophilic filaments in peripheral areas. Very little infiltration of inflammatory cells was observed around the colonies (Figure 2b). Based on these features, the diagnosis was infected keratocystic odontogenic tumor with actinomyces colonization.

Six months later, the surgeon reported normal healing. After 3 years, the patient did not show any pain or swelling, but he did not agree to undergo radiographic examination for further evaluation of other signs of recurrence. 

## Discussion

We presented a case of actinomycosis in association with recurrent KCOT. The infection was seen in a 17 year-old male. The previous studies have also reported a male tendency; although in older patients. In addition, the only similar reported case was found in a 39 year-old female.[7]

Trauma, dental and surgical procedures are some of the recognized factors that enhance the risk of actinomycosis. Actinomycosis may present as an acute inflammation similar to an abscess with pain.[1] In some cases, the infection causes a chronic inflammatory process with formation of granulation tissue or sinus tract.[2-6] The infection may be seen in association with osteomyelitis in area of trauma or surgical intervention or in association with odontogenic cysts, more commonly, radicular cysts that does not respond to routine root canal treatments.[8] The intra-osseous colonization of organism in the dentigerous cyst wall has been reported without any clinical and radiographic changes.[2] Other cases have been described in association with periapical lesions.[4] One case of infected odontogenic keratocyst with actinomycosis has also been observed in anterior part of mandible.[7] Actinomyces colonies are identified by typical morphology in routine hematoxylin and eosin sections.[6]

The filamentous bacteria create a radiating, basophilic center with an eosinophilic periphery; these colonies are surrounded by PMNs and granulation tissue and a fibrotic band encases this area.[9] Moreover; Periodic Acid Shift (PAS), Gram and methenamine silver stains clearly show the organisms.[9-10] In the present case, mild inflammation and fibrosis were found around the bacterial colonies. It has been suggested that presence of an inflammatory reaction and/or fibrosis in immediate proximity of bacterial colonies indicates a true infection and if these features are not found, actinomyces colonies are considered as inert floater bacteria.[4] Accordingly, the type of colonization in our case was similar to the second condition.

This study found these colonies to be in association with one out of two non-syndromic multiple KCOTs. KCOT tends to grow within the medullary cavity of bone and does not cause obvious expansion. Swelling may be seen in infected cysts.[11] Although, in our case, jaw expansion was found in the same location that the colonization was seen, it has been reported that the presence of bacterial colonies cannot predict the clinical course. Kaplaner *et al.* have demonstrated a direct correlation between the number, relative surface area, clinical course of infection and the heavier bacterial load which may result in a longer period of antibiotic treatment.[12] The present case showed a limited number of colonies without significant inflammatory cell infiltration around them and the swelling may be related to diffuse inflammation of the cyst wall rather than the actinomyces colonies. Previous studies reported a case of infected odontogenic keratocyst accompanied by pain in the anterior of mandible.[7]

In the present case, antibiotic therapy was not performed for actinomycosis and healing was found without any clinical complication. Although it has been proposed that all cases of true actinomycosis should be treated by surgical removal and antibiotic drugs for a long period,[7] surgery without additional antibiotic therapy was observed in some cases of periapical actinomyces.[11] Moreover, similar case of KCOT was also treated with curettage.[7] It was found that in patients that receive antibiotics without removal of the bacteria, recurrence may occur.[8] Therefore, it is important to perform surgical treatment with or without antibiotic therapy after diagnosis.

## Conclusion

In conclusion, actinomyces colonization may be found in association with recurrent pathologic lesions of the jaws such as developmental cysts following surgical treatments. However, it is possible to treat the lesion with standard surgery and without any additional treatment or considerations.  
